# Eph-ephrin Signaling Affects Eye Lens Fiber Cell Intracellular Voltage and Membrane Conductance

**DOI:** 10.3389/fphys.2021.772276

**Published:** 2021-11-25

**Authors:** Catherine Cheng, Junyuan Gao, Xiurong Sun, Richard T. Mathias

**Affiliations:** ^1^School of Optometry and Vision Science Program, Indiana University, Bloomington, IN, United States; ^2^Department of Physiology and Biophysics, State University of New York at Stony Brook, Stony Brook, NY, United States

**Keywords:** gap junction coupling, resistivity, connexin, aquaporin, EphA2, ephrin-A5

## Abstract

The avascular eye lens generates its own microcirculation that is required for maintaining lifelong lens transparency. The microcirculation relies on sodium ion flux, an extensive network of gap junction (GJ) plaques between lens fiber cells and transmembrane water channels. Disruption of connexin proteins, the building blocks of GJs, or aquaporins, which make up water and adhesion channels, lead to lens opacification or cataracts. Recent studies have revealed that disruption of Eph-ephrin signaling, in particular the receptor EphA2 and the ligand ephrin-A5, in humans and mice lead to congenital and age-related cataracts. We investigated whether changes in lens transparency in EphA2 or ephrin-A5 knockout (^–/–^) mice is related to changes in GJ coupling and lens fluid and ion homeostasis. Immunostaining revealed changes in connexin 50 (Cx50) subcellular localization in *EphA2*^–/–^ peripheral lens fibers and alteration in aquaporin 0 (Aqp0) staining patterns in *ephrin-A5*^–/–^ and *EphA2*^–/–^ inner mature fiber cells. Surprisingly, there was no obvious change in GJ coupling in knockout lenses. However, there were changes in fiber cell membrane conductance and intracellular voltage in knockout lenses from 3-month-old mice. These knockout lenses displayed decreased conductance of mature fiber membranes and were hyperpolarized compared to control lenses. This is the first demonstration that the membrane conductance of lens fibers can be regulated. Together these data suggest that EphA2 may be needed for normal Cx50 localization to the cell membrane and that conductance of lens fiber cells requires normal Eph-ephrin signaling and water channel localization.

## Introduction

The eye lens is a transparent, avascular, and ellipsoidal tissue in the anterior segment of the eye. It changes shape to allow fine focusing of light onto the retina to form a clear image. The bulk of the lens is composed of elongated fiber cells that are covered by a monolayer of epithelial cells covering the anterior hemisphere. Epithelial cells near the lens equator proliferate and differentiate into new generations of fiber cells, and the continuous addition of fiber cells cause life-long growth of the lens (Bassnett, 2002; Lovicu and Robinson, 2004). Lens fiber cells are connected by a network of large gap junction (GJ) plaques (Gong et al., 1997; Rong et al., 2002; Mathias et al., 2010; Cheng et al., 2015). The avascular lens is hypothesized to be supported by a microcirculation with an inward current at the anterior and posterior poles of the lens that is generated primarily by sodium as the ions enter the lens via the extracellular space between the cells (Mathias et al., 1997; Candia and Zamudio, 2002; Vaghefi et al., 2012). Sodium ions enter lens fiber cells by moving down the transmembrane electrochemical potential, and large GJ plaques facilitate the outflow of sodium ions toward the surface of the lens in the equatorial region (Baldo and Mathias, 1992). Sodium ions are actively transported out of the lens by Na^+^/K^+^-ATPase in the equatorial epithelial cells (Gao et al., 2000; Candia and Zamudio, 2002; Tamiya et al., 2003). The sodium current facilitates the circulation of fluids and other small molecules into the lens at the anterior and posterior poles and outflow of waste at the equator (Mathias et al., 1997; Merriman-Smith et al., 2003; Lim et al., 2005, 2006; Li et al., 2007; Gao et al., 2011; Candia et al., 2012; Vaghefi et al., 2012). Loss of connexin proteins lead to the formation of cataracts, defined as any opacity in the lens (Gong et al., 1997; White et al., 1998; Gao et al., 2004; Wang et al., 2009). During aging, decreased GJ coupling and microcirculation current has been linked to age-related nuclear cataracts (Gao et al., 2013).

Lens cells primarily utilize three isoforms of connexins. Lens epithelial cells express connexin 43 (Cx43) (or α1) and connexin 50 (Cx50) (or α8) while fiber cells use Cx46 (or α3) and Cx50 (Beyer et al., 1987; Gong et al., 1997; Rong et al., 2002). Embryonic lens fibers also express Cx23 (Puk et al., 2008), but it remains unclear whether Cx23 forms functional GJs (Sonntag et al., 2009). Six connexins oligomerize to form a connexon or hemichannel (Kumar and Gilula, 1996; Kar et al., 2012), and docking of two connexons from neighboring cells creates a GJ channel that allows the transfer of small molecules between the connected cells (Harris, 2007). Connexons can be formed by a mixture of different connexins (heteromeric) or be formed by just one type of connexin (homomeric) (Kumar and Gilula, 1996; Mathias et al., 2010), and GJs can be homotypic channels with two identical connexons composed of one type of connexin subunit, heterotypic channels with homomeric connexons each containing a different type of connexin or heterotypic channels with heteromeric connexons (Kumar and Gilula, 1996; Mathias et al., 2010). The composition of connexins in connexons and GJs determines the permeability of the channels.

Eph receptors are the largest class of receptor tyrosine kinases, and bidirectional signaling is mediated by the binding of Eph receptors to membrane-anchored ephrin ligands, which leads to forward signaling in the Eph-bearing cell and reverse signaling in the ephrin-bearing cell (Pasquale, 2008). Eph-ephrin signaling plays a role in cell-cell contact-dependent communication, cell-cell recognition events, including axon pathfinding, early segmentation and organ morphogenesis and cytoskeletal dynamics (Holland et al., 1996; Davy et al., 1999; Kullander and Klein, 2002; Himanen et al., 2007; Arvanitis and Davy, 2008). Our work and other recent studies show that loss of EphA2 or ephrin-A5 in the lens can causes cataracts and abnormal cell membranes, cytoskeletal networks, and fiber cell morphologies. In *EphA2* knockout (^–/–^ or KO) mouse lenses, mild nuclear cataracts were observed along with disrupted actin cytoskeleton, cell shape and organization of equatorial epithelial cells (Cheng et al., 2013), and misaligned and disorganized fiber cells (Jun et al., 2009; Cheng and Gong, 2011; Shi et al., 2012; Cheng et al., 2013; Zhou and Shiels, 2018). In contrast, *ephrin-A5*^–/–^ mouse lenses develop anterior cataracts or severe lens rupture depending on strain background (Cooper et al., 2008; Cheng and Gong, 2011; Biswas et al., 2016). In *ephrin-A5*^–/–^ mice in the C57BL6 background, anterior lens epithelial cells undergo epithelial-to-mesenchymal transition (EMT) caused by abnormal localization of E-cadherin and β-catenin (Cheng and Gong, 2011).

Since the loss of EphA2 leads to mild nuclear cataracts, we examined whether changes in GJ coupling could be contributing to cataractogenesis. Despite changes in the immunostaining pattern of Cx50, there is no obvious change in GJ coupling in *EphA2*^–/–^ lenses. We observe changes in intracellular voltage in *ephrin-A5*^–/–^ and *EphA2*^–/–^ lens fibers, and this change may be related to changes in the water channel, aquaporin 0 (Aqp0) protein distribution. These data suggest that Eph-ephrin signaling affects lens fiber cell membrane voltage, but that the cataracts in *EphA2*^–/–^ lenses are not due to changes in GJ coupling.

## Materials and Methods

### Mice

Mice were maintained in accordance with approved animal protocols (State University of New York at Stony Brook and Indiana University Bloomington Institutional Animal Care and Use Committees) and the National Institutes of Health guide for the care and use of laboratory animals. *Ephrin-A5*^–/–^ and *EphA2*^–/–^ mice were generated and maintained as previously described (Okabe et al., 1997; Frisen et al., 1998; Cheng and Gong, 2011; Cheng et al., 2013). All mice were maintained in the C57BL/6J background with wild-type *Bfsp2* (CP49) genes. Genotyping was performed by automated qPCR on toe or tail snips (Transnetyx, Cordova, TN, United States). Male and female littermates were used for experiments. We did not observe any obvious differences in lens phenotype between male and female mice.

### Lens Gap Junction Coupling and Membrane Voltage Measurements

Gap junction coupling conductance measurements were made as previously described (Gao et al., 2013; Cheng et al., 2015). The relationship of the “series resistance” to GJ coupling conductance is presented in Mathias et al. (1981), where the specialized electronics, necessary for wide bandwidth recordings in the lens, are also described. Control studies showing the relationship of the series resistance to GJ coupling conductance are reviewed by Mathias et al. (2010). In the current study, 3- and 12-month-old mice were used. Measurements were repeated on at least 6 lenses from 3 mice for each genotype and age. Due to partial dissection of the eyes for these experiments, images of the lenses were not obtained. Lenses without obvious severe cataracts were used for these experiments. Briefly, eyes were immediately enucleated from euthanized mice and placed in a Sylgard lined Petri dish filled with normal Tyrode solution containing 137.7 mM NaCl, 2.3 mM NaOH, 5.4 mM KCl, 2 mM CaCl_2_, 1 mM MgCl_2_, 5 mM HEPES, and 10 mM glucose (pH 7.4). After dissection to remove the cornea, iris, and optic nerve, the sclera was cut into 4 flaps, and the lens was mounted to the bottom of a chamber with a Sylgard base. The sample was mounted on the stage of the microscope, and the lens was maintained in normal Tyrode solution. One intracellular microelectrode was inserted into a fiber cell near the lens center where a wideband stochastic current was injected. A second intracellular microelectrode was placed at various depths between the periphery and center of the lens, and the intracellular voltage and lens impedance were recorded at each depth. The distances *r* (cm) from the center of a lens of radius *a* (cm) were recorded, and the impedance (induced voltage divided by injected current) was recorded using a Fast Fourier Analyzer (Hewlett Packard, Palo Alto, CA, United States). The radial intracellular resistance of GJs (*R*_*S*_) between the point of recording and the surface of the lens was estimated from the magnitude of the impedance at 1,000 Hz (Mathias et al., 1981). Four to five recordings were made in each lens from the voltage-recording intracellular microelectrode that was advanced radially into the lens. *R*_*S*_ values from at least 6 lenses of each genotype were pooled and curve-fitted using equations below:

$$w\,h\,e\,n\,b\leq r\leq a:R_{S}\,\left(r\right)=\frac{R_{D\,F}}{4\,\pi }\,\left(\frac{1}{r}-\frac{1}{a}\right)$$

w⁢h⁢e⁢n⁢ 0≤r≤b⁢RS⁢(r)=RD⁢F4⁢π⁢(1b-1a)+RM⁢F4⁢π⁢(1r-1b)

Mature fiber cells generally have increased intracellular resistivity (*R*_*MF*_, Ω⋅cm) compared to peripheral fibers (*R*_*DF*_, Ω⋅cm) with the change occurring abruptly at *b* = 0.85a, the site of transition from DF to MF where organelles are degraded and Cx46 and Cx50 proteins are C-termini cleaved. GJ coupling conductance (*G*) per area of cell-to-cell contact (S/cm^2^) is calculated by:

$$G_{D\,F}=\frac{1}{w\,R_{D\,F}}$$

$$G_{M\,F}=\frac{1}{w\,R_{M\,F}}$$

where *w* = 3 μm based on the radial spacing between GJ plaques. The intracellular voltage (*ψ_*i*_*) with respect to the bathing solution was recorded at each depth. In the lens, there is no single “resting voltage” as in isolated cells. The circulating current is associated with a radial gradient in the intracellular voltage, with a value of around −50 mV in central fiber cells to about −60 mV in surface cells. The transmembrane voltage is the difference in the intracellular and extracellular (between fibers) voltages, both of which vary with radial location, but we cannot measure the voltage in the tiny extracellular spaces, so we report only the intracellular voltage with respect to the bathing solution. Impedance studies at each depth were made to determine surface cell membrane capacitance (*C*_*S*_) and membrane conductance (*G*_*S*_), the fiber cell membrane capacitance (c*_*m*_*) and conductance (*g*_*m*_), and the effective extracellular resistivity (*R*_*e*_) between fiber cells were calculated based on an equivalent circuit model (Mathias et al., 1981) using the equations below. Note that *R*_*S*_(*r*) depends on radial position but not the sinusoidal frequency of the injected current, whereas *Z*_*L*_(*j*ω) is a property of membrane conductances and capacitances of all lens cells and varies with sinusoidal frequency but not location (see Figure 6 of Mathias et al., 2010, for typical data).

$$Z\,\left(j\,\omega \right)=R_{S}\,\left(r\right)+Z_{L}\,\left(j\,\omega \right)$$

$$Z_{L}\,\left(j\,\omega \right)=\frac{1}{4\,\pi \,a^{2}\,\left(Y_{S}\,\left(j\,\omega \right)+Y_{e}\,\left(j\,\omega \right)\right)}$$

$$Y_{S}\,\left(j\,\omega \right)=G_{S}+j\,\omega \,C_{S}$$

$$Y_{e}\,\left(j\,\omega \right)=\frac{\gamma }{R_{e}}\,\left(\coth\gamma \,a-\frac{1}{\gamma \,a}\right)$$

$$\gamma =\sqrt{R_{e}\,\frac{S_{m}}{V_{T}}\,\left(g_{m}+j\,\omega \,c_{m}\right)}$$

The surface of fiber cell membrane per unit volume of tissue is $\frac{S_{m}}{V_{T}}=6000\,{\text{cm}}^{-1}$, and *j*ω is the Fourier transform complex variable representing sinusoidal frequency.

### Lens Protein Extraction and WES Capillary-Based Westerns

Fresh lenses were dissected and collected from 6-week-old mice and stored at −80°C until homogenization and protein extraction as previously described (Cheng et al., 2018). Briefly, two lenses from each mouse were pooled into one protein sample, and there were at least three mice used for each experiment. Using a glass Dounce homogenizer, lenses were homogenized on ice in 250 μl of lens homogenization buffer [20 mM Tris, pH 7.4 at 4°C, 100 mM NaCl, 1 mM MgCl_2_, 2 mM EGTA, and 10 mM NaF with 1 mM DTT, 1:100 Protease Inhibitor Cocktail (P8430, Sigma-Aldrich) and 1 tablet of PhosStop per 10 ml buffer (04906845001, Roche) added on the day of the experiment] per 10 mg of lens wet weight. Protein samples were briefly sonicated and boiled for 5 min. Protein concentration was determined using Quick Start™ Bradford 1× Dye Reagent (Bio-Rad, Hercules, CA, United States) using manufacturer instructions. Equal amounts of protein were loaded for capillary-based (Western [WES]) immunoassay using a 12–230 kDa separation module kit according to manufacturer’s instructions (Protein Simple, San Jose, CA, United States) as previously described (Parreno et al., 2020). For each antibody, prior optimization experiments were run to determine the optimal protein and antibody concentrations. To detect Cx46 and Cx50 protein levels, we loaded 1 mg/ml of protein extracts onto WES plates and probed with rabbit polyclonal anti-Cx46 antibody [α3J, intracellular loop, 1:100, a generous gift from Dr. Xiaohua Gong (University of California, Berkeley) (Gong et al., 1998)] or rabbit polyclonal anti-Cx50 antibody [C-terminal, 1:100, a generous gift from Dr. Jose M. Wolosin (Mount Sinai School of Medicine) (Chang et al., 2002)]. To detect aquaporin 0 (Aqp0) protein levels, 0.12 mg/mL of protein extract was loaded onto WES plates and probed with rabbit polyclonal anti-Aqp0 antibody (1:50, AQP01-A, Alpha Diagnostic International, San Antonio, TX, United States). A Total Protein Separation module kit (Protein Simple) was used to detect the total protein amount for normalization. The data from WES plates are presented in electropherogram format and as virtual blots. Virtual blots convert electropherogram data into images of band densities similar to images of standard Western blots. The area under the peak of detected proteins is calculated and normalized to the total protein amount. Mean, standard deviation, and statistical significance (Student *T*-test, two-tailed between KO samples and respective controls) were calculated using Excel and graphed using GraphPad Prism 9. The proprietary matrix and running buffer for capillary electrophoresis differ from traditional SDS-PAGE gels and can lead to small changes in the apparent molecular weight of the detected peaks. The amount of SDS in the proprietary sample buffer (Hjemel and Chrambach, 1981; Rath et al., 2009) and the composition of the unique biotinylated molecular weight marker used for capillary electrophoresis can also influence the shift in apparent molecular weight (Sallantin et al., 1990). We only observe a single peak in each capillary, and this peak is close to the expected size of the protein that is being probed.

### Immunostaining of Frozen Sections

Freshly enucleated eyes from 6-week-old mice were fixed and sectioned as previously described (Cheng et al., 2016, 2018). Briefly, eyes were fixed for 4 h in 1% paraformaldehyde at 4°C. Samples were cryoprotected in sucrose and frozen in OCT medium (Sakura Finetek, Torrance, CA, United States) for sectioning. Twelve-micron-thick sections were then permeabilized, blocked, and stained as previously described (Nowak et al., 2009; Nowak and Fowler, 2012) using rabbit anti-Cx46 (1:200), rabbit anti-Cx50 (1:100), or rabbit anti-AQP0 (1:100) antibodies. Aqp0 detection with this antibody has been demonstrated previously in rat and mouse lens frozen sections with very low signal in the epithelium and high signal in the lens fibers (Grey et al., 2009; Petrova et al., 2015). Our immunostaining protocol may be causing slightly increased Aqp0 signal in lens epithelial cells compared to previous reports. Secondary antibodies were Alexa-488-conjugated donkey-anti-rabbit (1:200, 711-545-152, Jackson ImmunoResearch Laboratories, West Grove, PA, United States) or Alexa-647-conjugated donkey-anti-rabbit (1:200, 711-605-152, Jackson ImmunoResearch). Rhodamine phalloidin (1:100; Thermo Fisher Scientific, Waltham, MA, United States) was used to stain F-actin, and slides were mounted using Vectashield with DAPI (nuclei, Vector Laboratories, Burlingame, CA, United States). Images were collected using a Zeiss LSM800 confocal microscope. The epithelial cell thickness was used to identify cross-sectional tissue sections near the lens equator (Nowak et al., 2009; Cheng et al., 2016, 2018). Staining was repeated on at least 3 samples from different mice for each genotype, and representative data are shown. Images in the figures are from the same section going from peripheral to mature fiber cells, and these images were captured from neighboring depths without gaps or overlap using the motorized stage of the microscope for accuracy.

### Immunostaining Quantification

Aqp0 puncta/aggregate mean and max area measurements were conducted on 50 μm × 50 μm regions of interest (ROI) in the peripheral fiber cells (adjacent to the epithelial cell layer) and in the mature fibers (∼350 μm from the epithelial cell layer). Grayscale images of the Aqp0 staining signal were analyzed by Volocity 6.5 (Quorum Technologies, Puslinch, ON, Canada) using the Find Object function (threshold 10, minimum object size 0.2 μm^2^) and Separate Touching Objects module (object size guide 0.5 μm^2^). Mean and maximum Aqp0 puncta/aggregate areas were obtained from three different sections of each genotype. Mean, standard deviation, and statistical significance (Student *T*-test, two-tailed between KO samples and respective controls) were calculated and graphed using GraphPad Prism 9.

Aqp0 staining intensity measurements were conducted on 50 μm × 50 μm ROI in differentiating fiber cells (∼250 μm from the epithelial cell layer). Grayscale images of the Aqp0 signal were used, and mean gray values at the short and long sides of six cells were analyzed and averaged on each section using FIJI software (Schindelin et al., 2012). The average intensity ratio of the Aqp0 signal for each section is reported in the graphs. Mean, standard deviation, and statistical significance (Student *T*-test, two-tailed between KO samples and respective controls) were calculated and graphed using GraphPad Prism 9.

## Results

### Connexin 50 and Aquaporin 0 Localization Are Disrupted in EphA2^–/–^ Cortical Lens Fibers

**FIGURE 1:**
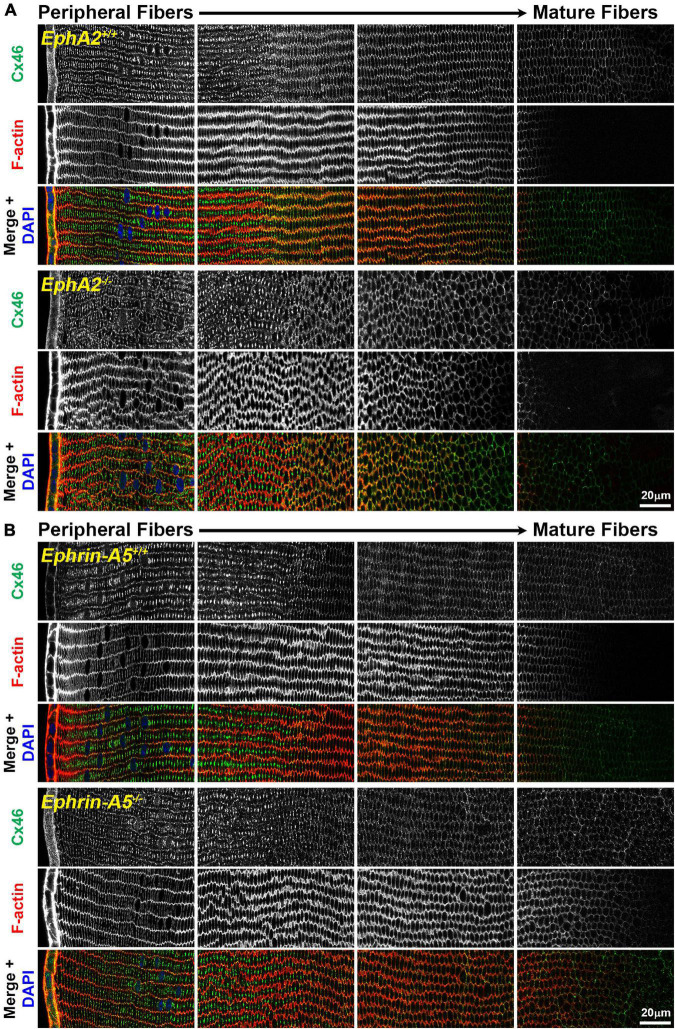
Connexin 46 (Cx46, green) and phalloidin (F-actin, red) staining in cross sections from control, *EphA2*^–/–^ and *ephrin-A5*^–/–^ lenses. Images are from the periphery to the inner mature fiber cells of the lens. **(A)** Cx46 is present at the cell membrane and is enriched on the long sides of peripheral differentiating fiber cells and becomes uniformly distributed around the cell membrane during maturation. The staining pattern is similar between control and *EphA2*^–/–^ lens fibers, despite the disorganization of *EphA2*^–/–^ cells. **(B)** Cx46 displays a similar localization pattern in peripheral and mature control and *ephrin-A5*^–/–^ lens fibers. Scale bars, 20 μm.

**FIGURE 2:**
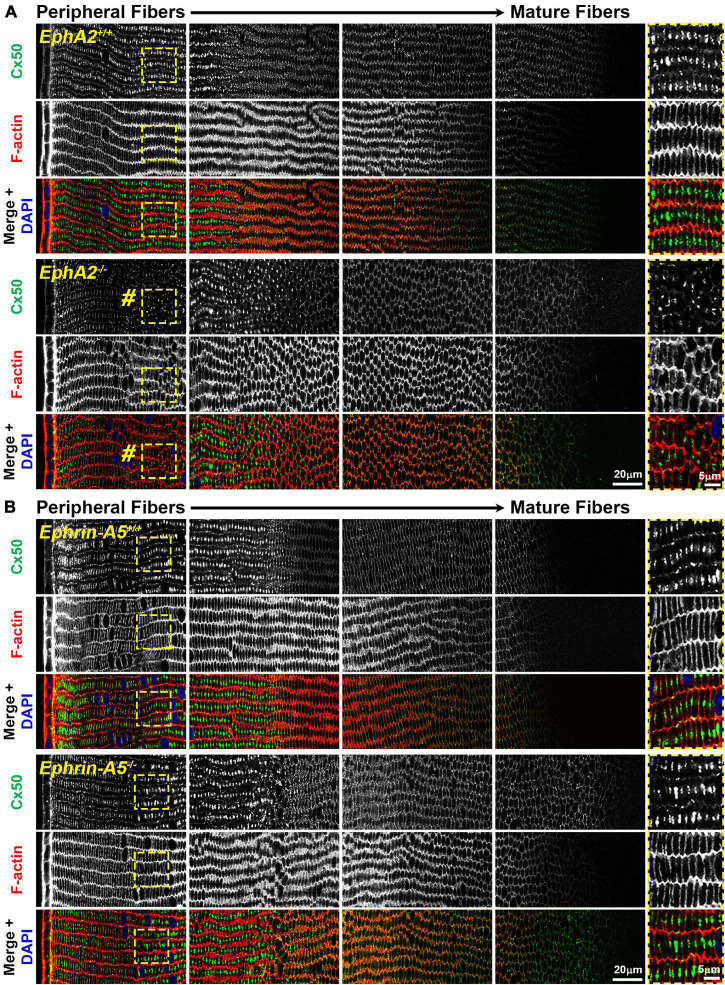
Connexin 50 (Cx50, green) and phalloidin (F-actin, red) staining in cross sections from control, *EphA2*^–/–^ and *ephrin-A5*^–/–^ lenses. Images are from the periphery to the inner mature fiber cells of the lens. **(A)** In control lens fibers, Cx50 is present at the cell membrane of peripheral cells and enriched in large plaques in the middle of the long sides of those cells. As fiber cells mature, Cx50 is distributed around the cell membrane with slight enrichment at the short sides. Unexpectedly, in *EphA2*^–/–^ peripheral lens fibers, there is a distinct region where the Cx50 signal is reduced at the cell membrane and is no longer in large plaques in the middle of the long side of the fibers (pound signs and high magnification panels on the right). As the cells mature, the Cx50 signal in *EphA2*^–/–^ fiber cells is comparable to the control. **(B)** Cx50 staining signals are comparable between control and *ephrin-A5*^–/–^ lens fibers in the peripheral and mature fibers. Scale bars, 20 and 5 μm.

While the mechanism for cataracts in humans with EphA2 mutations remain unknown (Shiels et al., 2008; Tan et al., 2011; Masoodi et al., 2012; Sundaresan et al., 2012), changes in ion/fluid homeostasis due to disruption of connexins (GJs) or aquaporins (water channels) can lead to nuclear cataracts (Gong et al., 1998; White et al., 1998; Rong et al., 2002; Wang et al., 2009; Gao et al., 2013; Kumari et al., 2017). Since mild nuclear cataracts are often present in *EphA2*^–/–^ lenses, we evaluated the localization of Cx46, Cx50, and Aqp0 by performing immunostaining in 6-week-old control, *ephrin-A5*^–/–^ and *EphA2*^–/–^ lens frozen sections. *Ephrin-A5*^–/–^ lenses were also evaluated in this study as a comparison for *EphA2*^–/–^ lenses and to learn more about whether these two molecules interact in subpopulations of cells in the lens. While Cx46 staining patterns were not significantly different between control and KO lens sections (Figure 1), immunostaining for Cx50 demonstrates that GJ plaques are reduced in peripheral *EphA2*^–/–^ fibers (Figure 2, pound signs and enlargements in dashed boxes). The Cx50 staining signal defect is restricted to peripheral *EphA2*^–/–^ lens fibers (Supplementary Figure 1). The normal F-actin staining signal in the *EphA2*^–/–^ lens section reveals that the change in the Cx50 signal in the periphery is not due to a sectioning or imaging artifact. The Cx50 staining signal was similar in other regions of the lens fibers of control and *EphA2*^–/–^ lenses. These defects were not detected in *ephrin-A5*^–/–^ lens sections (Figure 2).

**FIGURE 3:**
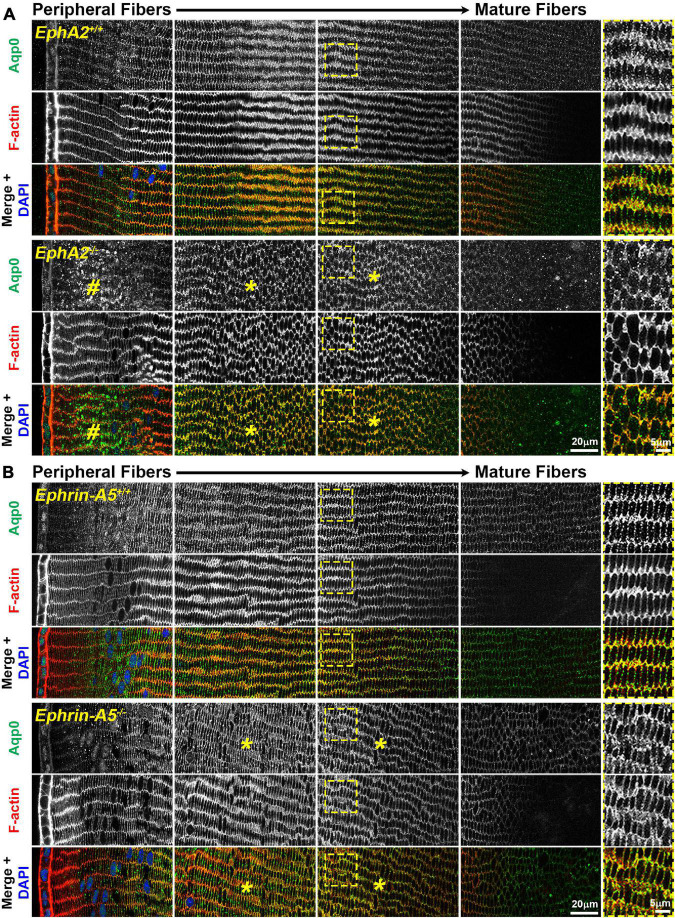
Aquaporin 0 (Aqp0, green) and phalloidin (F-actin, red) staining in cross sections from control, *EphA2*^–/–^ and *ephrin-A5*^–/–^ lenses. Images are from the periphery to the inner mature fiber cells of the lens. **(A)** In control peripheral lens fibers, Aqp0 is added to the cell membrane and evenly distributed around the membrane. As the cells mature, Aqp0 is enriched on the short sides of the fiber cells. In *EphA2*^–/–^ peripheral fiber cells, there are large puncta/aggregates of Aqp0 (pound signs), and as the cells mature, Aqp0 is distributed around the cell membrane without enrichment along the short sides of the cells (asterisks and high magnification panels on the right). **(B)** Compared to control fibers, Aqp0 distribution in differentiating and maturing *ephrin-A5*^–/–^ fibers is around the entire cell membrane (asterisks and high magnification panels on the right) while Aqp0 is enriched along the short sides of control lens fibers. Scale bars, 20 and 5 μm.

**FIGURE 4:**
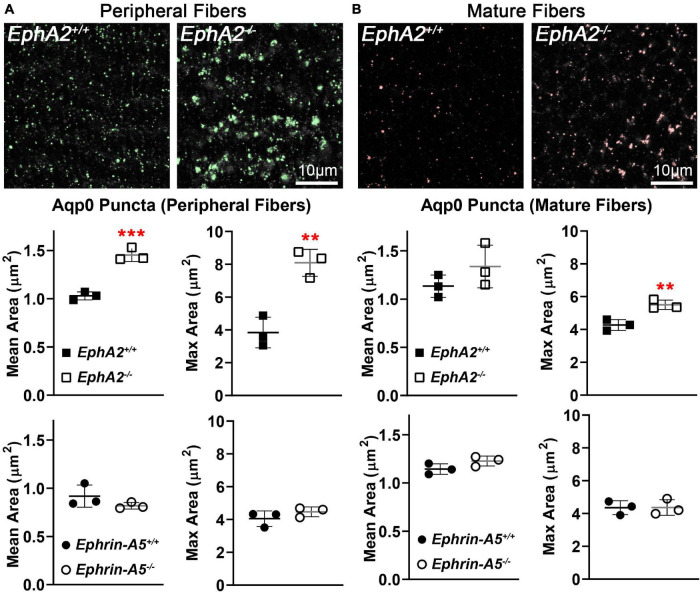
Quantification of Aqp0 puncta in peripheral and mature fiber cells from cross sections of control, *EphA2*^–/–^ and *ephrin-A5*^–/–^ lenses. **(A)** In control peripheral lens fibers, there are relatively small Aqp0 puncta, while in *EphA2*^–/–^ cells, there are much larger Aqp0 puncta/aggregates (highlighted in green). Quantification from three staining samples shows small Aqp0 puncta (mean and max puncta area, μm^2^) in *EphA2*^+/+^, ephrin-A5^+/+^ and *ephrin-A5*^–/–^ peripheral lens fibers. In contrast, Aqp0 puncta/aggregates are significantly larger (mean and max area) in *EphA2*^–/–^ peripheral lens fibers. **(B)** In mature *EphA2*^–/–^ lens fibers, larger Aqp0 puncta/aggregates are observed (highlighted in red). Quantification shows increased Aqp0 max puncta area in *EphA2*^–/–^ mature fibers. There are no changes in Aqp0 puncta found in *ephrin-A5*^–/–^ mature lens fibers. Scale bars, 10 μm. **, *p* < 0.01 and ***, *p* < 0.001.

**FIGURE 5:**
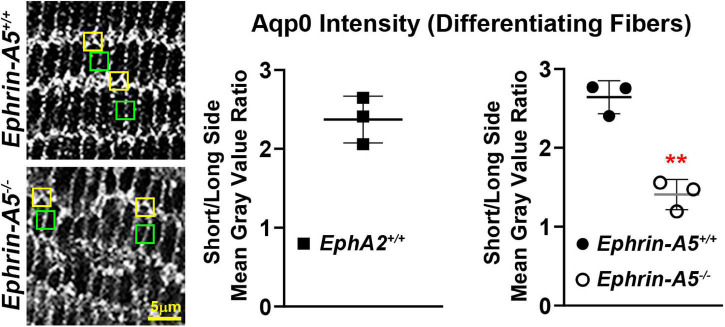
Comparison of the intensity of the Aqp0 immunostaining signal between the long and short sides of differentiating lens fiber cells. Quantification was performed by measuring the mean gray value of at the short side (yellow boxes) and the neighboring long side of the same cell (green boxes). Measurements were made in three different lens sections from each genotype. Immunostaining signal from *EphA2*^–/–^ mature lens fibers could not be measured as the cell shape is irregular, and the long and short sides of the cells could not be reliably distinguished. In control (*EphA2*^+/+^ and *ephrin-A5*^+/+^) mature lens fibers, the ratio of mean gray value (intensity) between the short and long side of the cell was over 2.5, indicating a stronger Aqp0 staining signal on the short side of the cell compared to the long side. In contrast, the ratio of intensity is just above 1 in *ephrin-A5*^–/–^, suggesting that Aqp0 staining intensity was similar between the short and long sides of these KO cells. Scale bar, 5 μm. **, *p* < 0.01.

Immunostaining for Aqp0 reveals that the water channel protein is abnormally aggregated in *EphA2*^–/–^ peripheral lens fibers (Figure 3, pound signs). There were also some larger Aqp0 puncta in the mature *EphA2*^–/–^ fiber cells. Quantification of the mean and maximum area of Aqp0 puncta/aggregates in peripheral fiber cells revealed that Aqp0 puncta/aggregates were increased in size (mean and max area) in *EphA2*^–/–^ lenses (Figure 4A). A similar analysis of mature fiber cells showed that Aqp0 puncta/aggregates had increased max area in *EphA2*^–/–^ lens sections (Figure 4B). Quantification did not reveal any changes in Aqp0 puncta in peripheral and mature *ephrin-A5*^–/–^ lens fibers. Interestingly, in mature *EphA2*^–/–^ and *ephrin-A5*^–/–^ lens fibers, Aqp0 is distributed evenly around the membrane unlike in control fibers where Aqp0 is enriched on the short sides of mature cells (Figure 3, asterisks and enlargements in dashed boxes). Aqp0 staining intensity (mean gray value) was measured and ratioed between the short and long sides of differentiating fibers in *EphA2*^+/+^, *ephrin-A5*^+/+^, and *ephrin-A5*^–/–^ lens sections (Figure 5). The Aqp0 intensity ratio could not be determined in *EphA2*^–/–^ lens fibers due to the irregular shape, leading to difficulties in reliably identifying the short vs. long sides of the cells. The ratio reveals that Aqp0 signals were more similar between the short and long sides of *ephrin-A5*^–/–^ differentiating lens fibers compared to that in control cells, suggesting a change in Aqp0 distribution around the cell membrane in KO lens fibers. These data suggest cell-cell adhesion defects and possibly an imbalance in lens fluid and ion transport in *EphA2*^–/–^ and *ephrin-A5*^–/–^ lenses.

### Loss of EphA2 or Ephrin-A5 Does Not Affect Aquaporin 0 and Connexin 50 Protein Levels

**FIGURE 6:**
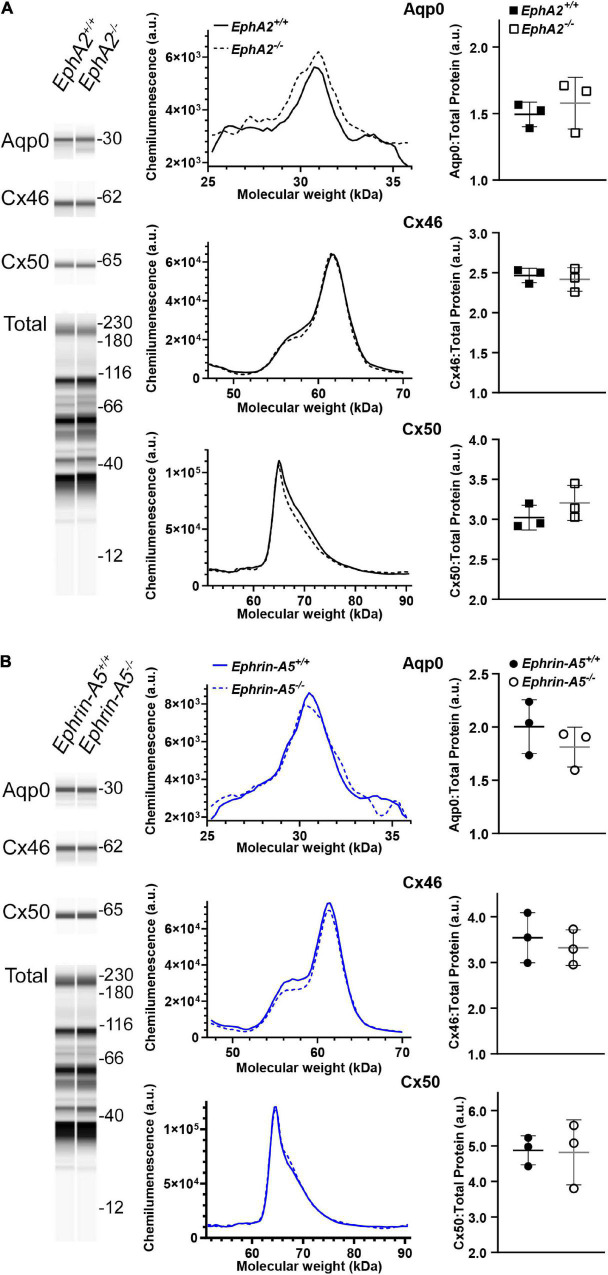
WES capillary-based Westerns for Cx46, Cx50, and Aqp0 in whole lens protein lysates from 6-week-old control, *EphA2*^–/–^ and *ephrin-A5*^–/–^ lenses. Representative gel bands for Aqp0 (30 kDa), Cx46 (60 kDa), and Cx50 (64 kDa) and total protein profiles (12–230 kDa) are shown in pseudo-lane views. **(A)** Representative electropherogram of Aqp0, Cx46, and Cx50 peaks are plotted for control and *EphA2*^–/–^ samples. Dot plots show the average and standard deviation of Aqp0, Cx46, and Cx50 protein levels normalized to the total protein. There is no difference detected between control and KO lenses in the protein amount for Aqp0, Cx46, and Cx50 (*p* > 0.3). **(B)** The same analysis was conduced for control and *ephrin-A5*^–/–^ lenses. There were no statistically significant differences in Aqp0, Cx46, or Cx50 protein levels detected.

Since Cx50 and Aqp0 localization is abnormal in *EphA2*^–/–^ lenses and Aqp0 distribution is also affected in *ephrin-A5*^–/–^ lenses, we performed quantitative Westerns of total lens homogenates from 6-week-old control, *EphA2*^–/–^ and *ephrin-A5*^–/–^ mice using the ProteinSimple WES capillary electrophoresis system. We showed the virtual lane view that is similar to traditional Western blot scans and is a representation of chemiluminescence peaks from each protein of interest and the total protein used for data normalization. The graphed electropherograms representing chemiluminescence peaks for each target protein were quantified for dot plots of protein amount normalized to total protein peaks. We did not detect any changes in Cx46, Cx50, or Aqp0 protein levels between control, *ephrin-A5*^–/–^ and *EphA2*^–/–^ lenses (Figure 6). These data suggest that Eph-ephrin signaling does not affect the overall amount of connexin proteins but may influence membrane localization and subcellular distribution of Cx50 and Aqp0.

### Disruption of Eph-Ephrin Signaling Does Not Alter Gap Junction Coupling in Lens Fibers, but Selectively Perturbs Intracellular Voltage and Membrane Conductance

**FIGURE 7:**
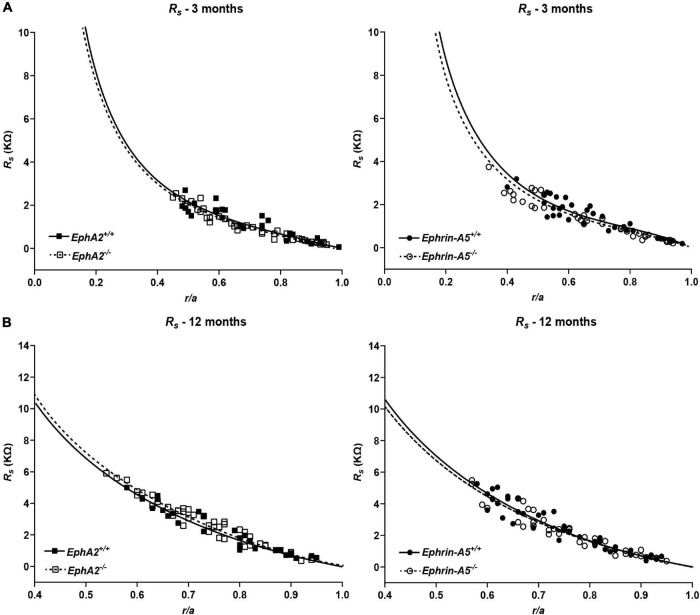
Series resistance measurements in lenses from 3- and 12-month-old control, *EphA2*^–/–^ and *ephrin-A5*^–/–^ mice. (A) Series resistance (*R*_*s*_) in lenses from 3-month-old *EphA2*^+/+^ (black squares with solid fit line), *EphA2*^–/–^ (open squares with dashed fit line), *ephrin-A5*^+/+^ (black circles with solid fit line), and *ephrin-A5*^–/–^ (open circles with dashed fit line) lenses as a function of distance from lens center (*r*/*a*), where *r* (cm) is actual distance and *a* (cm) is lens radius. There are no obvious differences in resistance between control and KO lenses (*n* ≥ 6). (B) Series resistance (*R*_*s*_) in lenses from 12-month-old *EphA2*^+/+^ (black squares with solid fit line), *EphA2*^–/–^ (open squares with dashed fit line), *ephrin-A5*^+/+^ (black circles with solid fit line), and *ephrin-A5*^–/–^ (open circles with dashed fit line) lenses as a function of distance from the lens center. There are no significant differences in resistance between control and KO lenses (*n* ≥ 6).

Since GJ plaque size and localization can affect fiber cell coupling and fluid and ion homeostasis (Cheng et al., 2015), we measured GJ coupling in 3- and 12-month-old control, *ephrin-A5*^–/–^ and *EphA2*^–/–^ lenses. Surprisingly, despite the change in Cx50 localization in peripheral fiber cells, there was no change in GJ conductance between lenses from 3-month-old control and *EphA2*^–/–^ mice (Figure 7 and Table 1). We also did not detect any changes in GJ conductance between lenses from 3-month-old control and *ephrin-A5*^–/–^ mice, consistent with our immunostaining and Western blotting data. A previous report showed that *EphA2*^–/–^ lenses develop age-related cortical cataracts that progress to whole cataracts and lens rupture (Jun et al., 2009). Thus, we also tested whether there were any GJ conductance changes in lenses from 12-month-old control, *ephrin-A5*^–/–^ and *EphA2*^–/–^ mice to determine whether GJ conductance changes could account for the age-related cataracts in *EphA2*^–/–^ lenses. We found no change in the GJ conductance between lenses from 12-month-old control and KO mice.

**TABLE 1:** Regional values of resistivity (*R*_*i*_) and normalized coupling conductance (*G*_*i*_) of four types of lenses at two ages in differentiating fibers (DF) and mature fibers (MF).

Genotype	Zone	Age	Resistivity (*R*_*i*_, KΩ -cm)	Conductance (*G*_*i*_, S/cm^2^)
*EphA2* ^+/+^	DF	3 month	4.48	0.74
*EphA2* ^–/–^	DF	3 month	4.13	0.81
*Ephrin-A5* ^+/+^	DF	3 month	5.55	0.60
*Ephrin-A5* ^–/–^	DF	3 month	5.13	0.65

*EphA2* ^+/+^	DF	12 month	11.25	0.30
*EphA2* ^–/–^	DF	12 month	11.37	0.30
*Ephrin-A5* ^+/+^	DF	12 month	10.46	0.32
*Ephrin-A5* ^–/–^	DF	12 month	9.79	0.34

*EphA2* ^+/+^	MF	3 month	2.80	1.19
*EphA2* ^–/–^	MF	3 month	2.64	1.26
*Ephrin-A5* ^+/+^	MF	3 month	3.00	1.11
*Ephrin-A5* ^–/–^	MF	3 month	2.77	1.20

*EphA2* ^+/+^	MF	12 month	11.20	0.30
*EphA2* ^–/–^	MF	12 month	11.27	0.30
*Ephrin-A5* ^+/+^	MF	12 month	11.46	0.29
*Ephrin-A5* ^–/–^	MF	12 month	10.68	0.31

**FIGURE 8:**
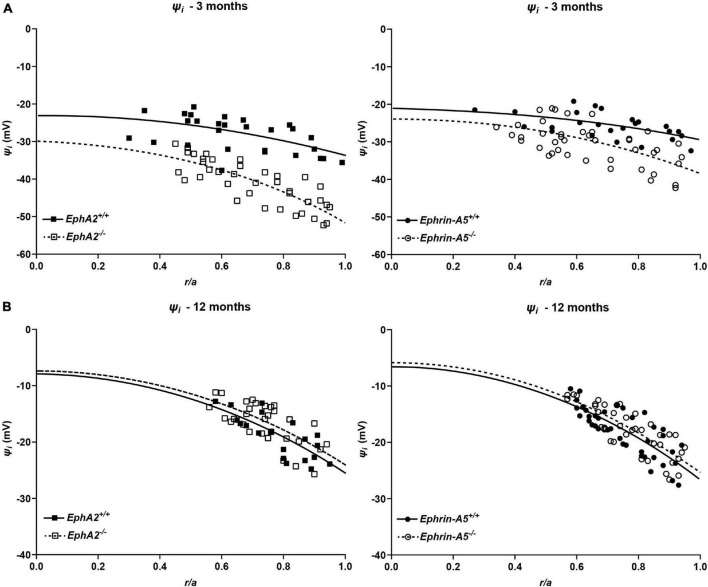
Intracellular voltage (*ψ_*i*_*) in lenses from 3- and 12-month-old control, *EphA2*^–/–^ and *ephrin-A5*^–/–^ mice. **(A)** Intracellular voltage in lenses from 3-month-old *EphA2*^+/+^ (black squares with solid fit line), *EphA2*^–/–^ (open squares with dashed fit line), *ephrin-A5*^+/+^ (black circles with solid fit line), and *ephrin-A5*^–/–^ (open circles with dashed fit line) lenses as a function of distance from lens center (*r*/*a*), where *r* (cm) is actual distance and *a* (cm) is lens radius. KO lenses were hyperpolarized when compared to controls (*n* ≥ 6). **(B)** Intracellular voltage in lenses from 12-month-old *EphA2*^+/+^ (black squares with solid fit line), *EphA2*^–/–^ (open squares with dashed fit line), *ephrin-A5*^+/+^ (black circles with solid fit line), and *ephrin-A5*^–/–^ (open circles with dashed fit line) lenses as a function of distance from the lens center. There are no significant differences in intracellular voltage between control and KO lenses (*n* ≥ 6).

**FIGURE 9:**
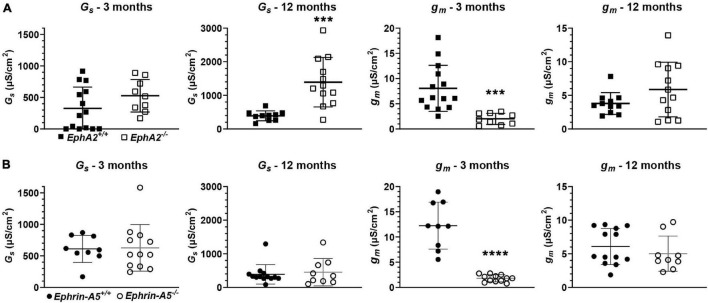
Surface conductance (*G*_*S*_) and mature fiber conductance (*g*_*m*_) in lenses from 3- and 12-month-old control, *EphA2*^–/–^ and *ephrin-A5*^–/–^ mice. **(A)** Mean and standard deviation of *G*_*S*_ and *g*_*m*_ in lenses from 3- or 12-month-old *EphA2*^+/+^ (black squares) and *EphA2*^–/–^ (open squares) mice. While *G*_*S*_ is comparable between lenses from young control and KO mice, *G*_*S*_ is elevated in lenses from 12-month-old *EphA2*^–/–^ mice. *g*_*m*_ is decreased in lenses from 3-month-old *EphA2*^–/–^ mice. **(B)** Mean and standard deviation of *G*_*S*_ and *g*_*m*_ in lenses from 3- or 12-month-old *ephrin-A5*^+/+^ (black circles) and *ephrin-A5*^–/–^ (open circles) mice. There is no obvious change in *G*_*S*_ in lenses from KO mice at either age or *g*_*m*_ in lenses from young KO mice. However, *g*_*m*_ is decreased in lenses from 12-month-old *ephrin-A5*^–/–^ mice. ***, *p* < 0.001; ****, *p* < 0.0001.

We also determined the intracellular voltage (*ψ_*i*_*) across control and *ephrin-A5*^–/–^ and *EphA2*^–/–^ lenses. In lenses from 3-month-old, *ephrin-A5*^–/–^ and *EphA2*^–/–^ mice, *ψ_*i*_* is significantly more negative when compared to littermate controls (Figure 8 and Table 2), indicating that the KO lenses are hyperpolarized compared to control lenses. In lenses from 12-month-old control, *ephrin-A5*^–/–^ and *EphA2*^–/–^ mice, the values of *ψ_*i*_* are very similar, showing that the membrane voltage defect is only present in lenses from young KO mice. We also measured the capacitance and conductance of surface and inner fiber cells as well as the effective extracellular and intracellular resistivity of fibers in lenses from 3- and 12-month-old control and KO mice. We did not find any statistical difference in surface or inner fiber capacitance or extracellular resistivity between control and KO lenses (data not shown), and those data were consistent with previous reports (Mathias et al., 1981; Martinez-Wittinghan et al., 2004). We did not observe any changes in conductance of surface cells (*G*_*s*_) in lenses from 3-month-old control, *ephrin-A5*^–/–^ and *EphA2*^–/–^ mice or in lenses from 12-month-old control and *ephrin-A5*^–/–^ mice (Figure 9). In lenses from 12-month-old *EphA2*^–/–^ mice, *G*_*S*_ was increased compared to controls. Unexpectedly, loss of either EphA2 or ephrin-A5 significantly decreased the conductance of fiber cell membranes (*g*_*m*_) in lenses from 3-month-old KO mice. The value of *g*_*m*_ was comparable between lenses from 12-month-old control and KO mice. Together these data suggest that while Eph-ephrin signaling does not affect GJ coupling between lens fibers, disruption of EphA2 or ephrin-A5 affects the conductance and intracellular voltage of fiber cells.

**TABLE 2:** Surface resting membrane voltage (*ψ_*m*_*) and the change in voltage between the surface and center (*Δψ_*m*_*) in four types of lenses at two ages.

	***Age***	***EphA2* ^+/+^**	***EphA2* ^–/–^**	***Ephrin-A5* ^+/+^**	***Ephrin-A5* ^–/–^**
***ψ _*m*_* (mV)**	3 month	–33.89	–51.23	–29.64	–38.37
*****Δ**ψ _*m*_* (mV)**	3 month	11.01	21.11	8.12	14.13

***ψ _*m*_* (mV)**	12 month	–25.60	–24.03	–26.55	–25.60
*****Δ**ψ _*m*_* (mV)**	12 month	17.82	16.72	20.04	19.80

## Discussion

In this work, we have shown that loss of EphA2 affects regional distribution, but not overall protein levels, of Aqp0 and Cx50. There are obvious aggregates of Aqp0 and loss of Cx50 staining in the same region of peripheral differentiating fiber cells in *EphA2*^–/–^ lenses. These defects are not present in *ephrin-A5*^–/–^ differentiating lens fibers. In mature fibers of both *EphA2*^–/–^ and *ephrin-A5*^–/–^ lenses, Aqp0 is evenly distributed around the cell membrane. In contrast, Aqp0 is normally enriched on the short sides of control mature fiber cells. Surprisingly, the change in Cx50 distribution in peripheral fibers does not result in defective fiber cell coupling in *EphA2*^–/–^ lenses. Previous data have shown that Cx50 contributes about half of *G*_*DF*_ (Gong et al., 1998), and thus, the disruption in the peripheral *EphA2*^–/–^ lens fibers could potentially affect GJ coupling. Other studies of connexin knockout/knockin lenses (reviewed in Mathias et al., 2010) have shown GJ coupling conductance varies directly with the level of expression of lens connexins. Here, we find the localization of Cx50 GJs is perturbed, but not the amount of expression. It appears that coupling depends primarily on expression and not on slight changes in localization. Similarly, since the loss of half the amount of Aqp0 in the lens does not affect *G*_*DF*_ (Kumari et al., 2017), the aggregation and mislocalization of Aqp0 in peripheral *EphA2*^–/–^ lens fibers does not affect the normal GJ coupling and conductance. The misalignment of fibers in *EphA2*^–/–^ lenses causes a misalignment of the large micron-size GJ plaques between the mature fibers, and thus, the disorganized GJ plaques do not affect resistivity. This data is consistent with our previous work that showed the size of Cx46 GJ plaques, rather than localization, affects lens fiber cell coupling (Cheng et al., 2015).

Our data suggest that in peripheral fiber cells, the localization of Aqp0 and Cx50 are not required for cell-cell coupling or communication, but rather the localization of these proteins may play a role in cell-cell adhesion. Cx50 and Aqp0 are known to be important for cell-cell adhesion between lens fibers (Costello et al., 1989; Zampighi et al., 1989; Michea et al., 1995; Palanivelu et al., 2006; Liu et al., 2011). The Cx50 defect in peripheral *EphA2*^–/–^ lens fibers may be downstream of Aqp0 aggregation dysfunction (Liu et al., 2011). In the lens, the mechanisms for GJ plaque and water channel assembly at the cell membrane are unknown. This is the first evidence that Cx50 GJ plaque and Aqp0 channel formation and maintenance at fiber membranes requires EphA2 signaling. Previous studies have linked Cx46 and Cx50 GJ properties to PI3K, MAPK, and TGFβ, downstream pathways that can be regulated by Eph-ephrin signaling (Shakespeare et al., 2009; Boswell et al., 2010; Martinez et al., 2015). We hypothesize that Eph-ephrin signaling is required for the recruitment of membrane-cytoskeletal components that are needed for GJ plaque and water channel assembly and/or stability in lens fibers. In other organisms and cell types, Eph-ephrin signaling can regulate GJ assembly and internalization by affecting the cell cytoskeleton or through growth factor signaling (Mellitzer et al., 1999; Miller et al., 2003; Davy et al., 2006; Ishii et al., 2011). In mouse inner ear cells, EphB2 regulates fluid homeostasis via interactions with PDZ proteins and Aqp1 (Cowan et al., 2000). In the lens, EphA2 interacts with PDZ protein, Dlg1, to regulate fibroblast growth factor (FGF) signaling (Lee et al., 2016). Alternatively, Ephs or ephrins in the lens may bind directly to Aqp0 or Cx50 to aid channel assembly and/or recycling. Further experiments will be required to determine how EphA2 and/or ephrin-A5 affect connexin and Aqp0 membrane trafficking and channel assembly in peripheral and mature lens fibers.

In our experiments, we find that 3-month-old *EphA2*^–/–^ and *ephrin-A5*^–/–^ lenses were hyperpolarized compared to controls. However, control lenses from 3-month-old mice of both mouse lines were depolarized compared to what has been measured in wild-type lenses from C57BL6 mice of a similar age (Baldo and Mathias, 1992; Mathias et al., 1997; Gong et al., 1998; Martinez-Wittinghan et al., 2004; Gao et al., 2013). Though our mouse lines were backcrossed to C57BL6 wild-type mice for many generations, these mice are not the same as the pure C57BL6 wild-type inbred strain. These data highlight the importance of utilizing littermate controls for experiments as we have done here, since even control mice may differ due to strain background and genetic drift. In the studies reported here, we compared control and KO lenses from littermates, and thus, we are certain the genetic backgrounds of the mice are the same. The only known difference between the control and KO lenses is the lack of EphA2 or ephrin-A5, suggesting the changes in KO lenses are related to disruption of Eph-ephrin signaling.

We only observe the change in the intracellular voltage in lenses from young KO mice, and the intracellular voltage is comparable between lenses from 12-month-old control and KO mice. The intracellular voltage of control lenses from 12-month-old mice was comparable to what was previously measured in wild-type lenses from 14-month-old C57BL6 mice (Gao et al., 2013). Though these data are unusual, they are self-consistent with our other measurements.

Intracellular voltage gradients have been hypothesized to be associated with circulations of sodium, potassium, and chloride ions within the lens (reviewed by Mathias et al., 1997). This voltage is not spatially uniform since it is associated with the current that flows intracellularly from cell to cell via GJs from the center of the lens to the surface. Intracellular voltage can therefore be affected by the loss of connexins due to genetic mutation or age (Baldo and Mathias, 1992; Gong et al., 1998; Gao et al., 2013) or changes in membrane conductance. As in other cells, in the lens, the intracellular voltage lies between K^+^ equilibrium potential (*E*_*K*_) and the Na^+^ equilibrium potential (*E*_*Na*_), and the intracellular voltage adjusts the Cl^–^ equilibrium potential (*E*_*Cl*_). In the lens, the weighting is a more complicated function of radial location (reviewed in Mathias et al., 1997).

The Na-conductance is in fiber cell membranes, whereas the K-conductance is in surface cell membranes (Mathias et al., 1997). In lenses from young control mice, *g*_*m*_ is much higher than that in lenses from KO mice, suggesting that Na-conductance is elevated. This may be the reason why control lenses from young mice were more depolarized than KO lenses. In lenses from 12-month-old control and KO mice, *g*_*m*_ and *ψ_*i*_* are comparable. These data suggest that Eph-ephrin signaling in the fiber cell domain declines with age, but depolarization due to age affects both control and KO lenses. Effectively, the age-dependent depolarization of lenses is independent of Eph-ephrin signaling. Gao et al. (2013) suggested that aging may lead to a decline of Na^+^/K^+^-ATPase activity that maintains the balance of sodium and potassium ions in the lens, thus leading to accumulation of sodium and depletion of potassium, and subsequently causes depolarization of the lens. The mechanisms by which Eph-ephrin signaling affects the intracellular voltage of the lens remains to be studied.

There is not much known about how *g*_*m*_ is established. There are no previous reports of changes in *g*_*m*_ in any other lenses. Our data are the first indication that this parameter can be regulated in the lens. Lower *g*_*m*_ would tend to decrease sodium ion flux into lens fiber cells and thus decrease water flow, but hyperpolarization of intracellular voltage would tend to do the opposite. Further experiments to measure the sodium ion concentration and hydrostatic pressure in *EphA2*^–/–^ and *ephrin-A5*^–/–^ lenses will be needed to determine whether hydrostatic pressure and sodium concentration changes could be correlated with the Aqp0 localization, or intracellular voltage and conductance changes seen in the *EphA2*^–/–^ and *ephrin-A5*^–/–^ mature fiber cells.

In lenses from 12-month-old, but not 3-month-old, mice, we found that loss of EphA2 but not ephrin-A5 is associated with a significant increase in surface cell conductance *G*_*S*_. However, there was no accompanying change in intracellular voltage. This contrasts with the reduction of *g*_*m*_ and hyperpolarization of *ψ_*i*_* in lenses from 3-month-old, but not 12-month-old, *EphA2*^–/–^ and *ephrin-A5*^–/–^ mice. First, while EphA2 signaling may be lost with age from the fiber cell membrane, it is still present and active in the surface cells of lenses from 12-month-old mice. If loss of EphA2 induces an increase in surface cell membrane K^+^-conductance, the effect on intracellular voltage would not be large since surface cell voltage is already close to *E*_*K*_. Another possibility is the increase in surface cell membrane Cl^–^-conductance (Mathias et al., 1997, 2007; Webb and Donaldson, 2009), which would also not significantly affect intracellular voltage since intracellular chloride is close to transmembrane equilibrium. Perhaps our methodology is not sensitive enough to detect the small voltage hyperpolarization in lenses from 12-month-old *EphA2*^–/–^ mice. Second, EphA2 signaling in lens surface cells of 12-month-old mice is not through the same pathway as ephrin-A5, since the surface conductance change is not present in *ephrin-A5*^–/–^ lenses. Taken altogether, these observations are puzzling, but the conductance and voltage data are internally consistent.

In summary, while there are no changes in GJ coupling in *EphA2*^–/–^ and *ephrin-A5*^–/–^ lenses, we have discovered that *g*_*m*_ and *G*_*S*_ of lens fibers can be regulated. These data point to important functions of EphA2 and ephrin-A5 in the lens that are beyond the known functions in cell morphology and cytoskeleton (Cooper et al., 2008; Cheng and Gong, 2011; Cheng et al., 2013; Son et al., 2013; Zhou and Shiels, 2018). There is very little known about how membrane conductance and resting voltage are established and maintained, and *EphA2*^–/–^ and *ephrin-A5*^–/–^ lenses provide a model to understand the mechanisms that determine these important cell parameters.

## Data Availability Statement

The original contributions presented in the study are included in the article/Supplementary Material, further inquiries can be directed to the corresponding author/s.

## Ethics Statement

The animal study was reviewed and approved by State University of New York at Stony Brook and Indiana University Bloomington Institutional Animal Care and Use Committees.

## Author Contributions

CC devised the main conceptual ideas, designed the study, and drafted the manuscript. All authors conducted the experiments, analyzed the data, and approved the final version of the manuscript.

## Conflict of Interest

The authors declare that the research was conducted in the absence of any commercial or financial relationships that could be construed as a potential conflict of interest.

## Publisher’s Note

All claims expressed in this article are solely those of the authors and do not necessarily represent those of their affiliated organizations, or those of the publisher, the editors and the reviewers. Any product that may be evaluated in this article, or claim that may be made by its manufacturer, is not guaranteed or endorsed by the publisher.

## Supplementary Material

Supplementary Figure 1 Connexin 50 (Cx50, green) and phalloidin (F-actin, red) staining in cross sections from control and *EphA2*^–/–^. These are low magnification images of the immunostained sections. In *EphA2*^–/–^ peripheral lens fibers, there is decreased Cx50 signal at the cell membrane (asterisk). The change in Cx50 staining signaling in the *EphA2*^–/–^ lens section is not due to staining artifacts or tissue section defects since the F-actin staining appears normal. Scale bar, 50 μm.
