# Supplementary figures and images for: Eph-ephrin Signaling Affects Eye Lens Fiber Cell Intracellular Voltage and Membrane Conductance
Source: Front Physiol. 2021 Nov 25;12:772276. doi: 10.3389/fphys.2021.772276 (PMC8656704; doi:10.3389/fphys.2021.772276)

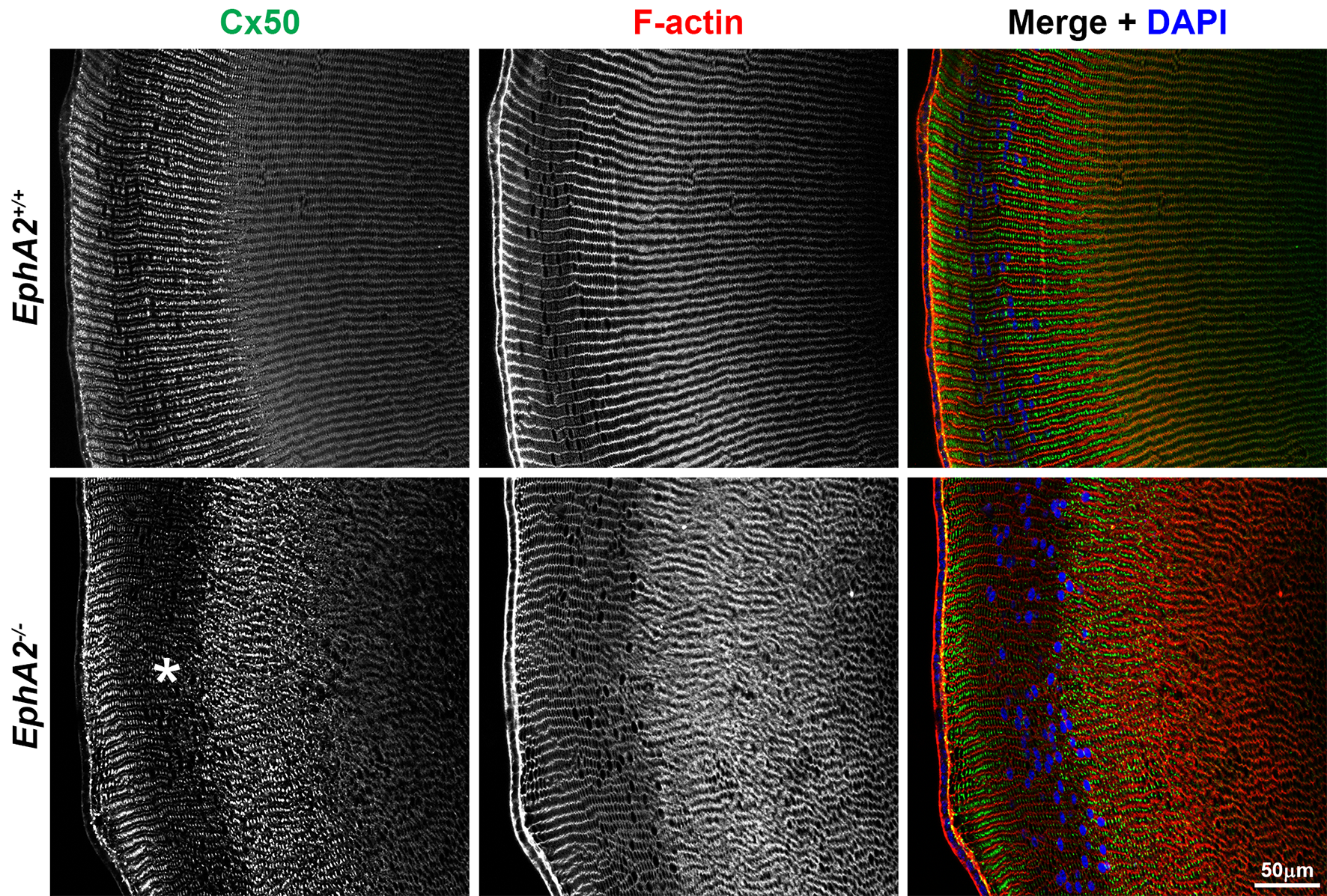

Supplement: Supplementary Figure 1 — Connexin 50 (Cx50, green) and phalloidin (F-actin, red) staining in cross sections from control and EphA2–/–. These are low magnification images of the immunostained sections. In EphA2–/– peripheral lens fibers, there is decreased Cx50 signal at the cell membrane (asterisk). The change in Cx50 staining signaling in the EphA2–/– lens section is not due to staining artifacts or tissue section defects since the F-actin staining appears normal. Scale bar, 50 μm. [file Image_1.TIF]
